# Amylopectin structure and crystallinity explains variation in digestion kinetics of starches across botanic sources in an in vitro pig model

**DOI:** 10.1186/s40104-018-0303-8

**Published:** 2018-12-29

**Authors:** Bianca M. J. Martens, Walter J. J. Gerrits, Erik M. A. M. Bruininx, Henk A. Schols

**Affiliations:** 1 Laboratory of Food Chemistry, Wageningen University and Research, Bornse Weilanden 9, 6708 WG Wageningen, The Netherlands; 2 Animal Nutrition Group, Wageningen University and Research, De Elst 1, 6708 WD Wageningen, The Netherlands; 3 Royal Agrifirm Group, Agrifirm North West Europe, Landgoedlaan 20, 7325 AW Apeldoorn, The Netherlands

**Keywords:** Amylopectin side chain distribution, Amylose, Granule diameter, In vitro digestion kinetics, Pores

## Abstract

**Background:**

Starch is the main source of energy in commonly used pig diets. Besides effects related to the extent of starch digestion, also several effects related to variation in digestion rate have recently been demonstrated in non-ruminants. Different rates of starch digestion in animals and in in vitro models have been reported, depending on the botanic origin of starch. Starches from different botanic sources differ widely in structural and molecular properties. Predicting the effect of starch properties on in vitro digestion kinetics based on existing literature is hampered by incomplete characterization of the starches, or by a selective choice of starches from a limited number of botanic sources. This research aimed to analyse the relationships between starch properties and in vitro digestion kinetics of pure starches isolated from a broad range of botanic origins, which are used in non-ruminant diets or have a potential to be used in the future. Therefore we studied starch digestion kinetics of potato, pea, corn, rice, barley, and wheat starches, and analysed the granule diameter, number of pores, type and amount of crystalline structure, amylose content and amylopectin side-chain length of all starches.

**Results:**

Multivariate analysis revealed strong correlations among starch properties, leading us to conclude that effects of most starch characteristics are strongly interrelated. Across all analysed botanic sources, crystalline type and amylopectin chain length showed the strongest correlation with in vitro digestion kinetics. Increased percentages of A–type crystalline structure and amylopectin side chains of DP 6–24 both increased the rate of digestion. In addition, within, but not across, (clusters of) botanic sources, a decrease in amylose content and increase in number of pores correlated positively with digestion kinetics.

**Conclusion:**

The type of crystalline structure and amylopectin chain length distribution of starch correlate significantly with digestion kinetics of starches across botanic sources in an in vitro pig model. Variation in digestion kinetics across botanic sources is not additively explained by other starch properties measured, but appears to be confined within botanical sources.

**Electronic supplementary material:**

The online version of this article (10.1186/s40104-018-0303-8) contains supplementary material, which is available to authorized users.

## Background

Starch is the main source of energy in commonly used diets for non-ruminants. Starches in those diets are of various botanic origin, which usually causes variation in digestion rate in the gastro-intestinal tract. Diverse effects related to variation in starch digestion kinetics have been demonstrated in pigs. For example, diets containing resistant starch (RS) or slowly digestible starch (SDS) affect feeding patterns [1] and energy partitioning [2] when compared to diets with rapidly digestible starch (RDS). Also an asynchrony between the rates of glucose- and amino acid absorption negatively affects protein utilization in pigs [3] and poultry [4].

Starch is composed of two types of polysaccharides: amylose, a linear α(1–4) linked glucan, and the much larger amylopectin, an α(1–4) linked glucan that contains around 5% α(1–6) linkages resulting in a branched molecule [5]. Starch normally contains about 20–30% amylose and 70–80% amylopectin, but amylose content can range from < 1% in waxy starches and > 70% in certain high amylose starches [6]. The branched amylopectin molecule contains regions with low and high levels of branches. In highly branched regions, side-chains of amylopectin are grouped, forming crystalline zones (clusters) [7]. Side chains of the amylopectin molecule can be divided in A, B, and C chains. C chains constitute the backbones of the amylopectin molecules, to which B-chains are linked that at the same time carry one or more branches. B chains are given additionally a number based on their participation in side chain clusters. B_1_ chains participate in one cluster, B_2_ and B_3_ chains participate respectively in two or three clusters. A chains are present at the outside of the branched molecule and have only an α(1–6) linkage to B_1_ chains [8]. Based on the cluster model of Robin et al. [9] and on the study of Hanashiro et al. [10], A chains are believed to correspond with side chains with a degree of polymerization (DP) of 6–12, B_1_ chains with DP 13–24, B_2–4_ chains with DP 25–36, and B_5-x_-and C-chains with DP > 36. Clustered amylopectin side chains and amylose chains are organized in the helix conformation that subsequently forms crystalline structures, which can be divided into three types: A, B and C. In A-type crystalline starch, glucose helixes are packed densely whereas B-type crystalline starch is packed less dense, leaving room for water molecules in between the branches. C-type crystalline starch consists of a combination of A- and B-type crystallinity [5]. During starch biosynthesis, starch is deposited in alternating amorphous and crystalline shells (growth rings), 100–400 nm thick [7], ultimately resulting in a water-insoluble granule [6]. The shape, size and distribution of granules varies highly between botanic sources [11]. Granules also vary in the level of porosity and can have openings (pores) on the surface of the granule [12].

Research has aimed to describe relationships between structural starch features and (in vitro) digestibility. The outcome of research, however, is influenced by the choice of starting material. For instance, when analysing purified starch samples that originate from the same botanic source, high levels of amylose correlate positively with the proportion of in vitro measured RS [13–16]. This is supported by several in vivo studies, which have shown a negative correlation between amylose content and the blood glucose response [17, 18] and consequently the glycaemic index [19]. Furthermore, amylopectin affects digestibility within botanic sources as well, as longer amylopectin side chains correlate with a slower digestibility [20, 21]. Across botanic sources, it is believed that a higher proportion of crystallinity correlates with more RDS, whereas a higher amorphous fraction results in a lower rate of digestion [14, 22, 23], even though amorphous and crystalline regions are assumed to be digested simultaneously [24]. Due to the water molecules present in B- and C-type starch crystals, these starches are usually digested slower than the A-type starch [24, 25]. The size of the starch granule is also believed to correlate negatively with the rate of starch digestion across botanic sources. Smaller granules are digested faster than bigger ones, which is generally believed to be caused by a larger surface area on which enzymes can act [26, 27]. Lastly, the presence of pores is proven to affect starch digestion; due to these channels, enzymes are supposed to digest starch granules from the inside out, leading to a more rapid digestion [12, 28].

Estimating the contribution of each distinct characteristic to starch digestion kinetics is hampered by inherent combinations of these starch characteristics within each botanic source. For example, large granules are usually digested slower than small granules, but often consist of slowly digestible B-type crystals [27]. The same goes for amylose content and crystalline structure, as high amylose cereal starches are mostly reported to consist of C- or B-type crystalline structure, in contrast to A-type crystalline structure, which is typical for low amylose cereal starch [29–31]. Furthermore, amylopectin molecules with longer chains are correlate with more B-type crystals [20]. Also correlations between amylose content and granule size [32] and amount of crystalline structure [14] have been reported before.

Quantifying the influence of each individual starch characteristic based on literature is extremely difficult [33]. By relating all characteristics to the in vitro digestion kinetics of those starches, we aimed to identify, across botanic sources, the starch characteristics that are affecting starch digestion most in starches relevant for non-ruminant feed production. In this study we measured the following starch characteristics: type and amount of crystalline structure, amylose content, amylopectin chain length, granule size and the number of pores of starches from a wide range of botanic sources. The selected botanic sources are commonly used in animal feeds, or have a potential for future use. The purpose of this study was to correlate intrinsic starch properties to starch digestion kinetics. Consequently, we selected purified starches to eliminate effects of other feed related components.

## Materials and methods

**Pure starches** from different botanic origins were selected to cover maximum relevant variation in amylose content, granular size, proportion and type of crystallinity, and the presence of pores. 15 different starches were used, of which one starch was additionally sieved into 5 fractions, creating a total of 20 samples. Rice starches Remyline AX-DR (*waxy rice*), Remy B7 (*rice A*) and Remy B (*rice B*) were kindly provided by the Beneo group (Tienen, Belgium). Nastar yellow pea starch (*pea A*) was obtained from Cosucra group (Perq, Belgium) and wheat starch (*wheat*) was obtained from Fluka Biochemika (Buchs, Switzerland)*.* Corn starches M03401 (*corn A*) and M04201 (*waxy corn*) were kindly provided by Cargill B.V. (Vilvoorde, Belgium), high amylose corn starch Hylon V (*high amylose corn A*) was obtained from Ingredion (Westchester, IL, USA), and barley starch (*barley*) was kindly provided by Altia Corporation (Helsinki, Finland). Native potato starch, Eliane 100 (*waxy potato*), and experimental Heat Moisture Treated potato starch (*HMT potato*), based on native potato starch, were kindly provided by Avebe (Veendam, The Netherlands). Regular corn starch (*corn B*), high amylose Amylomaize (*high amylose corn B*), and regular pea starch (*pea B*) were obtained from Roquette (Nord-Pas-de-Calais, France). All starch samples were stored at room temperature until analysed. All enzymes and chemicals were purchased from Sigma Aldrich (Saint Louis, MO, USA) unless stated otherwise.

**Granule size distribution** was determined as the mean of 5 measurements (maximum standard deviation of the mean diameter was 0.41 μm) with a Mastersizer3000 (Malvern Instruments, Malvern, U.K.). Potato starch samples were fractionated using a vibratory sieve shaker (AS200 digit; Retsch GmbH & Co., Haan, Germany) with demineralized water as washing liquid. Size fractions were air-dried overnight at 40 °C. Potato starch was separated into five fractions: smaller than 20 μm, 21–32 μm, 33–53 μm, 54–71 μm, and 72–109 μm, of which the granule size distribution was determined as described above.

**Starch morphology** was determined with a Scanning Electron Microscope (SEM) (Magellan 400, FEI, Eindhoven, The Netherlands). Dry starch granules were attached on sample holders using carbon adhesive tabs (EMS, Washington, USA) and sputter coated with 15 nm tungsten (EM SCD 500, Leica, Vienna, Austria). Granules were analysed with a field emission SEM with SE detection at 2 kV. Starch morphology was studied at 1,000 times magnification and starch surfaces were studied at 25,000 times magnification. All samples were measured at the Wageningen Electron Microscopy Centre and from each sample at least 20 individual granule surfaces were studied. To ensure the analysis of a homogenous sample, individual granules were selected in such a way that granules from each diameter were represented in accordance with their relative abundance as measured with the Mastersizer.

**X-ray diffraction** (XRD) was used to identify the crystalline structure of starch samples. Wide angle X-ray scattering (WAXS) powder diffractograms were recorded on a Bruker Discover D2 diffractometer (Bruker corporation, USA) in the reflection geometry in the angular range 4–33°(2θ), with a step size of 0.02°(2θ) and an acquisition time of 2.0 s per step. The diffractometer was equipped with an LINEXEYE™ Silicon-strip detector, which had a 4°-5° active area. The Co Kα1 radiation (λ = 1.7902 Å; X-ray tube is air cooled) from the anode, generated at 30 kV and 10 mA, was monochromatized using a Ni filter. The diffractometer was equipped with a 1 mm divergence slit and a 0.5-mm knife edge above the sample stage, which enabled accurate measurements from 4° 2θ upwards. The proportion of crystallinity was determined by subtracting the background from the WAXS pattern of a sample. Intensities are expressed as relative where the intensity of the peak at diffraction angle 20.0°(2θ) is set to 1.0, as this peak showed the highest intensity in both A- as B-type of starch. Samples that contained a mixture of typical A- and B-type crystalline diffraction spectra were modelled (with a least-squares error fit procedure) to deduce the exact proportions of A- and B-type crystallinity.

**Amylose content** of starch samples was determined according to the amylose/amylopectin assay procedure of the supplier (Megazyme, Wicklow, Ireland), in which amylose was separated from amylopectin by Concanavalin A lectin. Amylose was enzymatically hydrolysed and glucose recovery was determined by a glucose oxidase peroxidase reaction. All samples were analysed in triplicate (maximum standard deviation of 0.8%).

**Total starch content** of each sample was determined according to the total starch assay procedure from Megazyme by subsequently washing the starch with ethanol (part e of the assay procedure supplied by Megazyme), dissolving the starch in KOH (part c of the assay procedure supplied by Megazyme) and finally enzymatically hydrolysing the starch (part a of the assay procedure supplied by Megazyme). Glucose was determined with a glucose oxidase peroxidase reaction. The total starch content was solemnly used as a correction factor in the in vitro digestion assay. All samples were analysed in triplicate (maximum standard deviation of 1.1%).

**Amylopectin chain length** was determined after starch samples were debranched with 0.32 mL isoamylase (500 units/mL, Megazyme) per gram starch at pH 4 for 17 h at 50 °C, after starch samples were dissolved in boiling water. Debranched samples were analysed on ICS5000 High Performance Anion Exchange Chromatography system with Pulsed Amperometric detection (HPAEC-PAD) (Dionex Corporation, Sunnyvale, CA, USA) equipped with a CarboPac PA-1 column (ID 2 mm × 250 mm) and a CarboPac PA guard column (ID 2 mm × 25 mm). The flow rate was set at 0.3 mL/min. The two mobile phases were (A) 0.1 mol/L NaOH and (B) 1 mol/L NaOAc in 0.1 mol/L NaOH and the column temperature was 20 °C. The elution profile was as follows: 0–50 min 5–40% B, 50–65 min 40–100% B, 65–70 min 100% B, 70–70.1 min 100–5% B and finally column re-equilibration by 5% B from 70.1 to 85 min. The injection volume was 10 μL. Glucose was used to quantify concentrations of side-chains with DP up to 5 (although this fraction made up < 0.1% of the amylopectin molecule) and maltohexaose was used to quantify side-chains with DP > 5. All samples were analysed in duplicate and the average amylopectin chain length per sample had a maximum standard deviation of 0.7 DP. To facilitate the comparison of starch samples, amylopectin side-chains were clustered into four categories, DP 6–12, DP 13–24, DP 25–36, and DP >  36, in analogy to the cluster model of Robin et al. [9]. For Pearson’s correlation procedure and PCA, variations in side-chain length of amylopectin were summarized in the ratio between short (DP 6–24) to long (DP > 36) side-chains. “Short” chains reflect A and B amylopectin side-chains that are involved in only 1 cluster, whereas “long” chains reflect B and C chains involved in 5 or more clusters, respectively [9, 10].

**In vitro starch digestion kinetics** were determined with a digestion method described by Englyst et al. [34] and Van Kempen et al. [35]. Briefly, 500 mg of starch was incubated with pepsin (P-7000) in a hydrochloric acid solution (0.05 mol/L), containing guar-gum and 50% saturated benzoic acid at pH 3 and 39 °C for 30 min, followed by incubation in a sodium acetate buffer (0.5 mol/L) containing porcine pancreatin (P-7545), amyloglucosidase (I4504) and invertase (A7095) at pH 6 and 39 °C for 360 min. In comparison to the assay described by Van Kempen et al. [35], samples were incubated in a head-over-tail mixing device (8 r/min) located in an oven. Furthermore, glucose concentrations were measured in smaller aliquots in a 96-well plate by using a glucose oxidase peroxidase assay (GOPOD, Megazyme). All samples were analysed in triplicate (maximum standard deviation of 7% glucose release per time point).

Although multiple equations can be used to model in vitro starch digestion, a relative simple model was chosen to enable further statistical analysis. This resulted in the use of a modified version of the Chapman-Richards model as previously described by van Kempen et al. [35](Eq. 1):

1 $$ starch\ hydrolysis= plateau\ast \left[1-\exp \left(-\frac{K/100}{Plateau}\ast 100\ast time\right)\right] $$

The *starch hydrolysis* is expressed as % of starch in sample, *plateau* is the maximum amount of glucose released during digestion, which is converted to starch (as % of sample weight) by multiplying with factor 0.9. *K* is the rate of glucose release corrected for plateau effects (as % of starch hydrolysed to glucose per minute). The model was fitted to the data using the average of at least triplicate observations. The *K* and plateau value of each starch sample were estimated by nonlinear regression procedures (proc NLIN, SAS, version 9.3, SAS Institute, Cary, USA). For estimation of the plateau value, a boundary was included forcing the estimation to be ≤100%. The model was also fitted after the plateau value was fixed to 100%, but the fit of this model was less good, as for some starches the estimated plateau value was far below 100%.

**Correlation coefficients** between measured starch properties were generated using Pearson’s correlation procedure (proc CORR) in SAS. Correlations with *P* ≤ 0.05 were taken as statistically significant and 0.05 < *P* ≤ 0.1 as a tendency for significance.

**Principal Component Analysis** (PCA) was conducted using the factor procedure in SAS on the starch characteristics, to examine whether variation in these starch characteristics could be summarized in the estimation of principle components (PC’s) that are uncorrelated. After extraction, PC’s were scaled by their standard deviations (square roots of associated Eigenvalues) and subjected to orthogonal rotation (varimax) to obtain independent factors. The amount of variation in starch digestion kinetics explained by each independent PC was analysed with Pearson’s correlation procedure.

## Results

### Characterization of starch

To make a comparison between starches from different botanic sources and their effect on in vitro starch digestion kinetics, 20 starches from different botanic sources (potato, pea, corn, rice, barley, and wheat) were analysed on several characteristics regarding the molecular and granular structure of the starches.

**Fig. 1:**
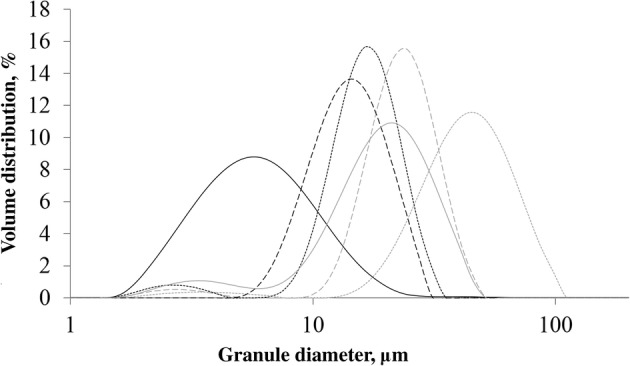
Granule diameter distribution of rice B, corn A, barley, wheat, pea A, and potato  starch

**The average granular diameter** of the starches used in this study ranged from 6 to 92 μm (Table 1). In general, rice starch had the smallest granules, followed by cereal starches (of which corn had the smallest granules and wheat the largest) and pea starch, whereas potato starch consisted of the largest granules. As shown, barley, wheat, pea A, and potato starch had a bimodal granule distribution that consisted of a small fraction of granules with a diameter between 1 and 7 μm and a much larger fraction of granules with a diameter > 7 μm (Fig. 1). Such a bimodal distribution was also seen for waxy potato, HMT potato, corn B, high amylose corn A, high amylose corn B, and pea B starch, but not for the three rice starches, corn A, and waxy corn starch (results not shown). Granule size distribution showed that sieving of potato starch successfully separated smaller granules from larger ones (size distribution not shown, average size in Table 1).

**Table 1:** Intrinsic properties of all analysed starch samples

Origin	Sample	Amylose content, %	Granule diameter, μm	Crystal content, %	Percentage A-type crystals	Number of pores, 100 μm^2^	St.dev. number of pores, 100 μm^2^	Side-chain length (DP) amylopectin					
								Average, mol%	6–12, mol%	13–24, mol%	25–36, mol%	> 36, mol%	Short:Long
Potato	Regular	18	45.8	30.0	0.0	0.1	0.3	31	4.6	22.1	14.1	59.2	0.45
Potato	Sieved (< 20)	15	18.9	30.5	0.0	0.0	0.0	31	4.3	22.4	14.6	58.8	0.45
Potato	Sieved (21–32)	18	30.8	31.5	0.0	0.0	0.0	31	4.3	22.2	14.3	59.2	0.45
Potato	Sieved (33–52)	18	49.7	31.2	0.0	0.0	0.0	31	4.6	22.5	13.8	59.1	0.46
Potato	Sieved (53–71)	18	71.2	30.6	0.0	0.2	0.4	31	4.7	22.8	13.9	58.7	0.47
Potato	Sieved (72–109)	18	92.2	22.7	0.0	0.2	0.4	29	5.3	25.1	13.9	55.7	0.55
Potato	Waxy	0	45.4	33.0	20.2	0.0	0.0	30	5.2	22.7	14.8	57.3	0.49
Potato	HMT	11	46.7	23.4	42.4	0.0	0.0	31	4.7	22.3	15.0	58.1	0.46
Pea	Regular A	27	24.7	27.3	73.4	0.0	0.0	26	6.8	29.7	20.2	43.2	0.85
Pea	Regular B	27	25.0	25.2	68.4	0.0	0.0	26	6.4	30.9	18.9	43.8	0.85
Corn	Waxy	0	16.0	28.2	100.0	36.7	25.2	23	9.6	35.3	17.9	37.3	1.20
Corn	Regular A	19	15.0	21.5	100.0	28.6	24.9	23	9.8	35.4	17.1	37.6	1.20
Corn	Regular B	19	14.6	28.2	100.0	28.6	41.9	23	10.3	36.1	17.2	36.4	1.28
Corn	High amylose A	46	13.8	28.0	41.0	3.5	8.7	32	4.5	21.5	13.6	60.4	0.43
Corn	High amylose B	55	11.0	33.7	41.3	3.3	9.9	33	3.9	18.8	13.8	63.5	0.36
Barley	Regular	20	17.3	28.3	100.0	1.1	2.0	23	7.7	29.1	20.5	42.7	0.86
Wheat	Regular	23	20.6	25.5	100.0	0.0	0.0	23	9.9	31.2	18.8	40.2	1.02
Rice	Waxy	1	8.3	27.1	100.0	13.0	26.0	23	11.1	33.2	15.1	40.6	1.09
Rice	Regular A	16	6.6	24.2	100.0	21.7	72.7	24	9.3	35.1	14.2	41.4	1.07
Rice	Regular B	14	6.2	28.5	100.0	10.8	29.3	26	7.7	27.3	17.4	47.7	0.73

**Fig. 2:**
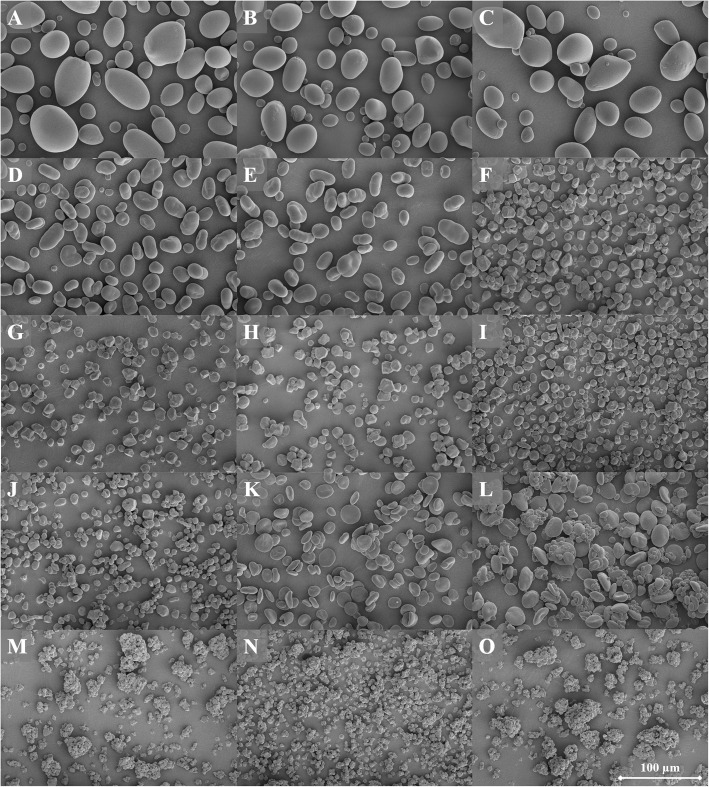
SEM pictures of the morphology of all starch samples (magnitude 1,000 times). A = regular potato, B = waxy potato, C = HMT potato, D = pea A, E = pea B, F = waxy corn, G = corn A, H = corn B, I = high amylose corn A, J = high amylose corn B, K = barley, L = wheat, M = waxy rice, N = rice A, O = rice B

**Starch morphology** was visualized by scanning electron microscopy (SEM), illustrating both variation in granular size, size distribution, and shape (Fig. 2). All varieties of potato and pea starch granules were large and round, whereas pea starch was more bean-shaped. All corn starches contained a mixture of squared and round granules, whereas barley starch had more disk-shaped granules. Wheat starch granules were roundly shaped and rice starch contained small, square shaped granules. Some starches seemed to stick together, which was especially clear for wheat and rice starches. The variation identified in granule size with SEM was comparable with the measured granule size distribution.

**Fig. 3:**
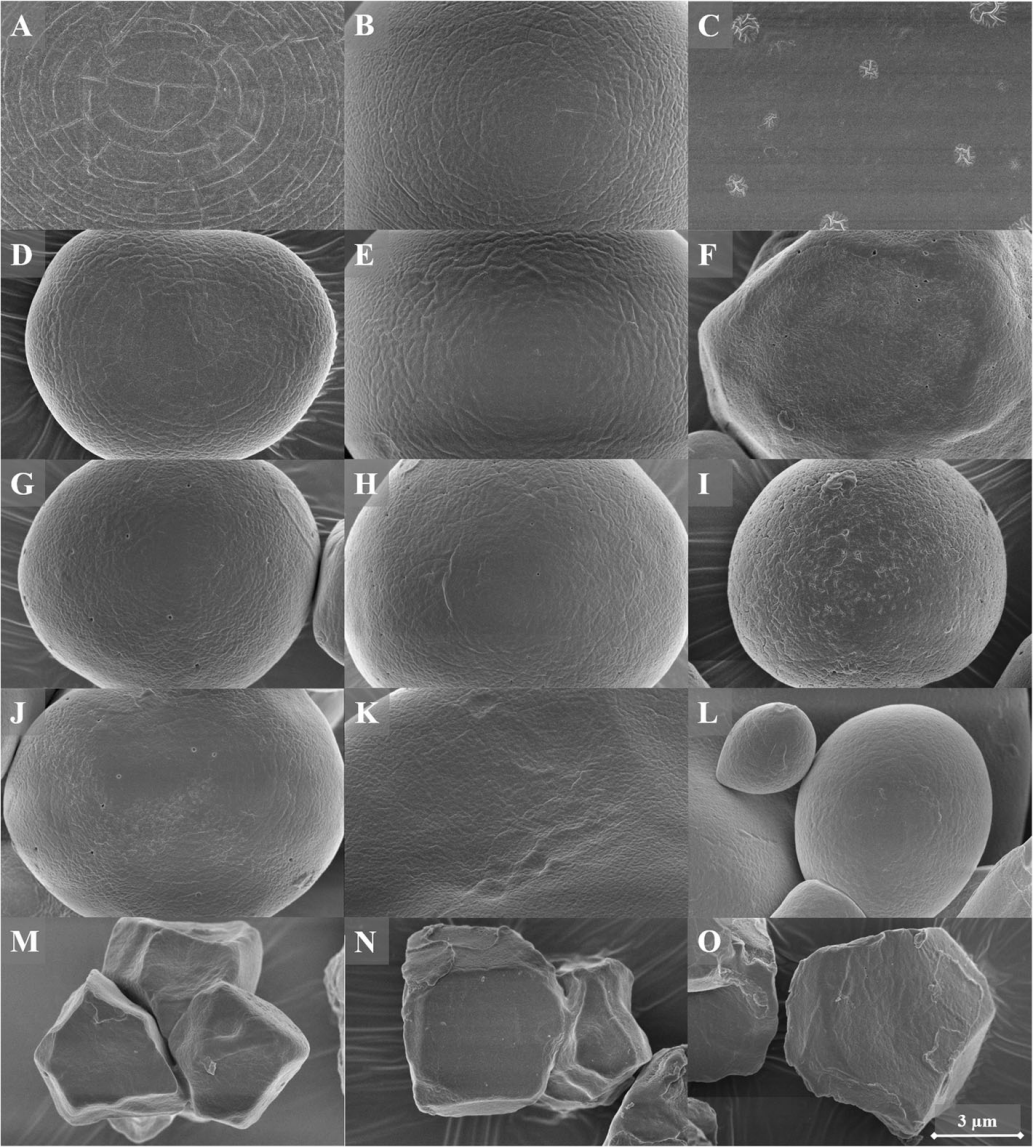
SEM pictures of the surface of all starch samples (magnitude 25,000 times). A = regular potato, B = waxy potato, C = HMT potato, D = pea A, E = pea B, F = waxy corn, G = corn A, H = corn B, I = high amylose corn A, J = high amylose corn B, K = barley, L = wheat, M = waxy rice, N = rice A, O = rice B

**Starch surface and presence of pores** was studied with SEM, visualising that all varieties of potato and pea starch had granules with a smooth surface without pores (Fig. 3). The smoothness of the surface of corn starch depended on the type of corn starch. Regular corn starches (corn A and B) had smooth granules, with small, round pores. Waxy and high amylose corn starches had granule surfaces that were less smooth, more porous and granules had more and irregular shaped pores. Barley starch had a smooth surface without pores, but merely scratches on several granules. Wheat starch had the smoothest surface of all starches, with no pores, cavities or irregularities. All varieties of rice starch had a smooth surface, with several small and round pores. The average number of pores (expressed per 100 μm^2^ of surface area, Table 1) enables quantitative comparison amongst samples. Most pores were observed in corn and rice samples, whereas starch from potato, pea, barley, and wheat all had none or a very limited number of pores.

**Fig. 4:**
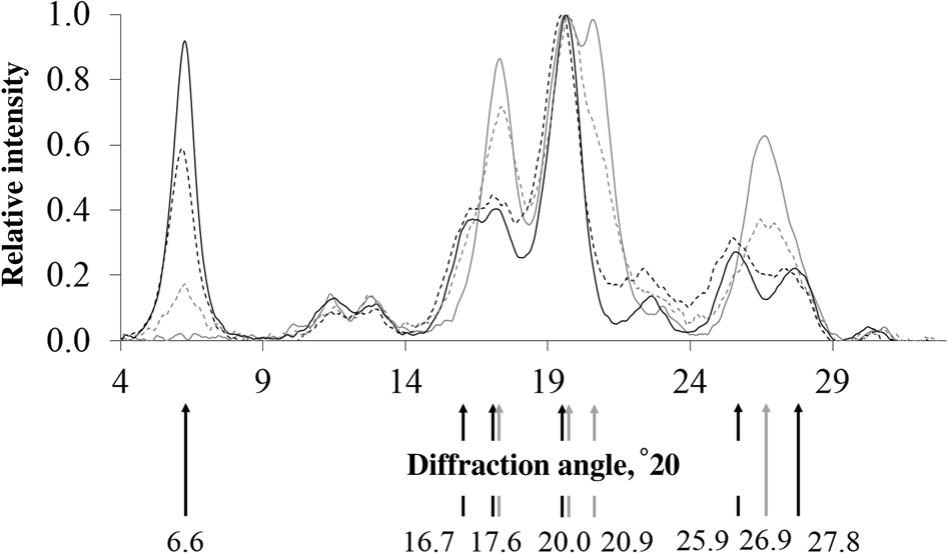
Wide-angle X-ray diffraction patterns for a typical type B crystalline starch (potato) ––, a typical type A crystalline starch (waxy rice)  , a typical type Ca crystalline starch (pea B), and a typical type Cb crystalline starch (waxy potato) --. Arrows indicate peaks that are typical for B-crystalline starch (➙) or for A-crystalline starch (), below which the corresponding diffraction angles are shown

**Crystalline structure** was studied with XRD. X-ray diffraction patterns of several starch samples are shown (Fig. 4) with arrows indicating peaks that are typical for A-type or B-type crystalline starch [36]. X-ray diffraction patterns of waxy corn, corn A, corn B, barley, wheat, waxy rice, rice A, and rice B starches contained solemnly representative peaks of A-type crystals and were therefore characterized as 100% A-type crystalline starch (Table 1). Potato starch contained only representative peaks for B-type crystalline starch. Some starch samples contained representative peaks of both types of crystallinity in different intensities resulting in the so-called C-type crystalline starch. This type of crystallinity can be close to B-type crystalline starch (type Cb) as observed for waxy potato, HMT potato, and high amylose corn, or closer to A-type crystalline starch (type Ca), as observed for both pea starches. Diffraction patterns of a typical B-type crystalline starch (potato) and a typical A-type crystalline starch (waxy rice) were used to calculate the exact proportion of A-type crystallites in each starch sample (Table 1).

**The relative amylose content** of the starches ranged from 0% for waxy samples (waxy rice, waxy corn, and waxy potato) to 55% for high amylose corn starch (Table 1).

**Fig. 5:**
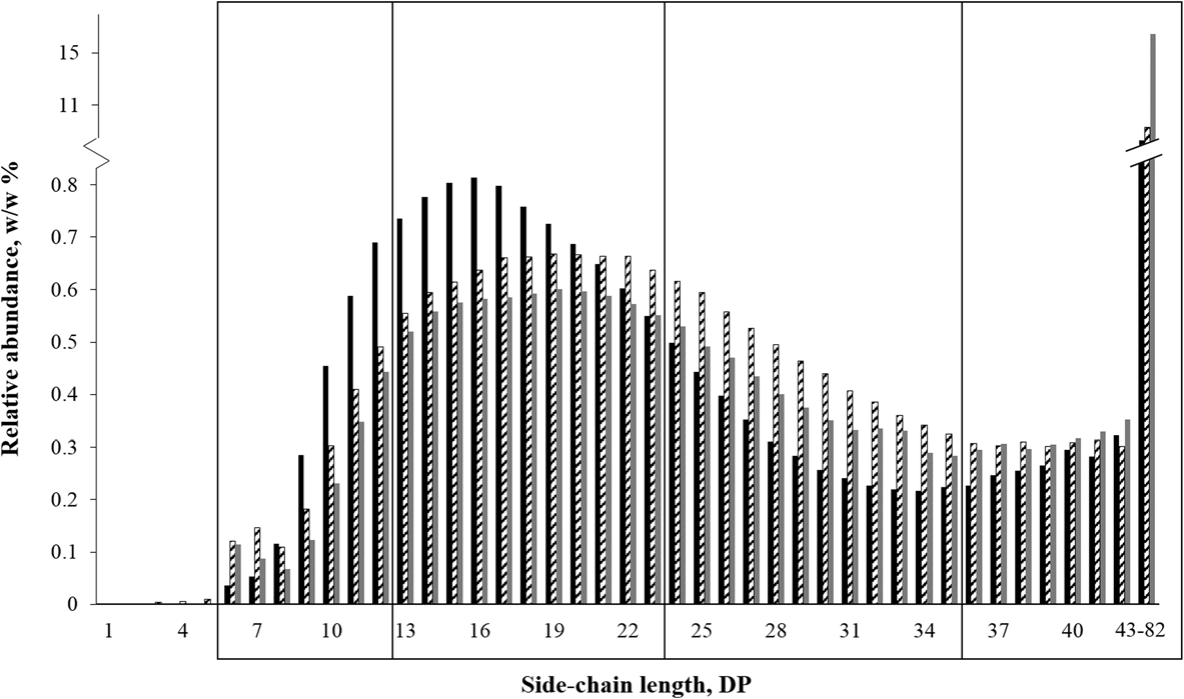
Amylopectin side-chain length distribution of rice A ■, pea A  and potato  starch. Clusters of side-chains are indicated with black boxes

**Amylopectin side-chain length distribution** ranged generally from short side-chains identified in cereal starches to long side-chains for potato starches; pea starches had an intermediate side-chain length distribution (Fig. 5). Concentrations of individual branches could be calculated up to DP 42, as chains with a larger DP could not be separated with the method used. Therefore, concentration of chains larger than DP 42 are presented as the sum of all soluble chains with DP > 42.

### In vitro digestibility

**Fig. 6:**
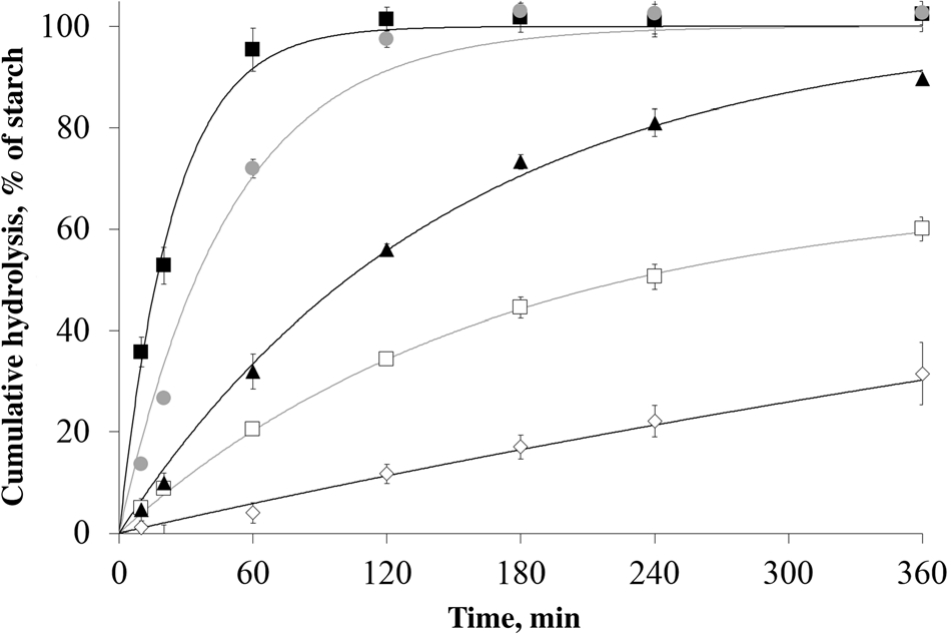
In vitro digestion kinetics for cereal starch (wheat ■, corn B , and high amylose corn B □), legume starch (pea B ▲), and tuber starch (potato ). Symbols indicate the average of in triplicate measured values, lines represent the first-order kinetic model fitted to these data

Digestion kinetics of all starches during incubation with porcine pancreatin, amyloglucosidase and invertase was measured over time and used to fit a first order kinetics model (Table 2 and Additional file 1: Table S1). The average glucose recovery per time point was used to fit the model. In vitro cumulative starch hydrolysis and the estimated model are illustrated (Fig. 6) for wheat being the most rapidly digestible starch tested, for potato as most resistant starch and for three intermediate digestible starch samples; corn, high amylose corn, and pea. The K value ranged from 0.08%/min for the more resistant types of starch, to 4.19%/min for rapidly digestible types of starch. Using data up to 360 min of digestion, the plateau value was estimated at > 95% for most starch samples except for waxy potato (40%), both high amylose corn starches (63, 67%), and pea A starch (93%).

**Table 2:** *K* and plateau values for all analysed starch sources, as estimated with Eq. 1

Origin	Sample	*K*, %/min	Plateau, %^a^	Residual sum of squares
Potato	Regular	0.10	100.0	11.8
Potato	Sieved (< 20)	0.14	100.0	7.9
Potato	Sieved (21–32)	0.10	100.0	7.6
Potato	Sieved (33–52)	0.08	100.0	31.7
Potato	Sieved (53–71)	0.08	100.0	2.3
Potato	Sieved (> 71)	0.09	100.0	1.4
Potato	Waxy	0.13	41.4	1.1
Potato	HMT	0.41	100.0	11.3
Pea	Regular A	0.67	92.7	74.2
Pea	Regular B	0.68	99.9	24.7
Corn	Waxy	2.55	100.0	44.7
Corn	Regular A	1.89	100.0	197.5
Corn	Regular B	2.03	100.0	168.7
Corn	High amylose A	0.41	63.1	14.5
Corn	High amylose B	0.40	67.3	6.9
Barley	Regular	3.22	100.0	95.5
Wheat	Regular	4.15	100.0	47.7
Rice	Waxy	4.29	100.0	5.6
Rice	Regular A	1.60	95.5	27.0
Rice	Regular B	3.06	95.6	69.0

^a^ Limited to a maximum of 100%

### Interrelationships among starch properties and digestion kinetics

Prior to correlating the structural starch properties with in vitro digestion kinetics, interrelations among the measured starch properties were examined and illustrated with PCA (Table 3). The first Principal Component (PC1), explaining 55% of the variance in starch properties, consisted of high loadings of 4 of the 6 parameters, namely granule diameter (− 0.85), pores (0.74), type of crystalline structure (0.93), and side-chain length of amylopectin (0.86), illustrating the interrelationships of these parameters among the analysed starch sources. The amylose content was the only parameter with a high loading (0.91) on PC2, explaining 22% of the variation. The proportion of crystalline structure is loaded comparably strong, but in opposite directions, on PC1 and PC2 (− 0.48 vs 0.41 respectively).

**Table 3:** Rotated factor pattern, eigenvalues and proportion variance explained of principle components in multivariate analysis

	PC1	PC2
Eigenvalues	3.30	1.29
Proportion variance explained	0.55	0.22
Loading of variables		
Granule diameter	−0.85	−0.46
Number of pores	0.74	−0.31
Crystal content	−0.48	0.41
Percentage A-type crystals	0.93	−0.12
Amylose content	−0.10	0.91
Ratio short:long amylopectin side-chains	0.86	−0.40

The potential of PC1, PC2 and each of the individual starch properties to explain variation in the rate of starch degradation is presented in Table 4. Of the two PC’s, only PC1 has a significant correlation with the rate of in vitro starch digestion (*r* = 0.67, *P* = 0.0061). This indicates that PC1, combining granule diameter, pores, type of crystalline structure, and the side-chain length distribution of amylopectin, is associated with variation in starch digestion rate. This association is less strong for PC2, mainly reflecting the effect of the amylose content. As the proportion of crystalline structure is loaded on both PC1 and PC2 equally, but with loadings below 0.5, this property appears to explain little variation in digestion kinetics.

**Table 4:** Pearson correlation coefficients for starch properties and digestion kinetics, of all analysed (non-sieved) starch samples^a^

		PC1	PC2	Amylose content	Granule diameter	Crystal content	% A-type crystals	Number of pores	Side-chain length amylopectin, DP					*K*
									6–12	13–24	25–36	> 36	Short:Long^b^	
PC1		X	0.00	−0.10	−0.85**	− 0.48*	0.93**	0.74**	0.85**	0.84**	0.36	−0.82**	0.86**	0.67**
PC2			X	0.91**	−0.46	0.41	−0.12	− 0.31	−0.39	− 0.43*	−0.14	0.40	−0.39	− 0.17
Amylose content				X	−0.24	0.18	−0.24	−0.36	− 0.48*	−0.45*	− 0.12	0.43	− 0.42	−0.37
Granule diameter					X	0.12	−0.74**	−0.50*	− 0.56**	−0.48*	− 0.13	0.48*	− 0.51*	−0.57**
Crystalline content						X	−0.49*	−0.31	− 0.45*	−0.56**	− 0.26	0.53*	− 0.51*	−0.25
% A-type crystals							X	0.57**	0.88**	0.84**	0.56**	−0.88**	0.86**	0.81**
Number of pores								X	0.65**	0.69**	−0.01	−0.59**	0.70**	0.33
Side chain length amylopectin, DP	6–12								X	0.93**	0.40	−0.92**	0.95**	0.81**
	13–24									X	0.49*	−0.98**	0.99**	0.60**
	25–36										X	−0.65**	0.49*	0.42
	> 36											X	−0.98**	−0.68**
	Short:long												X	0.65**
*K*														X

^a^ ** indicates a significant correlation (*P* ≤ 0.05), * indicates a tendency for a significant correlation (0.05 < *P* ≤ 0.10)

^b^ “Short” refers to amylopectin side-chains with DP 6–24 and “long” refers to amylopectin side-chains with DP> 36

Although 4 of 6 parameters have high loadings on PC1, the combination of parameters unexpectedly does not explain more variation in *K* (*r* = 0.67, *P* = 0.0061) than some of the parameters individually, such as the type of crystalline structure (*r* = 0.81, *P* = 0.0003) and the fraction of short (DP 6–12) amylopectin side-chains (*r* = 0.81, *P* = 0.0002).

## Discussion

Previous research has focussed on the contribution of starch digestion to dietary energy supply, but currently also the effects of variation in starch digestion kinetics have become more clear [1–4]. Properties related to the molecular and granular structure of starch cause variation in starch digestion kinetics [13, 14, 16, 21]. However, quantifying the contribution of every unique property is complicated by inherent combinations of properties within starches originating from a similar botanic source [14, 29–32]. Previous work showed that it is complicated to estimate the effect of each individual starch characteristic across botanic sources based on literature [33]. Therefore, this research aimed to identify, across botanic sources, the starch characteristics that are affecting starch digestion most in starches that are relevant for feed production for monogastric animals.

### Starch properties

Most of the measured starch properties are close to values that were published before [6, 10, 11, 30, 37–43], except the lack of bimodal size distribution in waxy corn and corn A, which is presumable the result of differences in isolation procedures between starch wholesalers. The low number of pores observed in barley and wheat starch partly contradicts previous literature [12, 44], in which more pores were observed for both cereals. This difference in pore abundancy may be related to heterogeneity of the analysed starches and to differences in the varieties analysed in our study compared with previous studies. In this study, a large number of individual granules were studied from a homogenous sample, providing a reliable insight in the number of pores.

### Starch digestion kinetics

The inevitable consequence of a model with only two parameters, is an imperfect fit of data for some starch sources (Table 2, Fig 6). However, it is a necessity to use the same, relatively simple model for all sources in order to relate the rate of digestion to starch properties. For some starches, the extent of starch digestion did not reach the estimated plateau value within 360 min of incubation and the plateau value was estimated at values below 100%. To obtain more insight in the biological meaning of the estimated plateau value, a prolonged incubation (*t* = 24 h) was performed with a selection of those starches, namely high amylose corn A and pea A starch. During this prolonged incubation the extent of starch digestion exceeded the estimated plateau value (towards 84.1% and 100% respectively). This indicates that the estimated plateau value is required for a good fit of data, but does not necessarily reflect the asymptotic maximum glucose release for all starch samples, as stated by van Kempen et al. [45] and Englyst et al. [34]. Therefore the rate of starch digestion, but not the extent, was used to study correlations between digestion kinetics and starch properties.

### Interrelationships among starch properties and digestion kinetics

As illustrated with PCA, variation in starch properties could be summarised in two independent factors, but neither factor additively explained variation in digestion kinetics. To understand the contribution of each distinct starch property to the variation observed in starch digestion kinetics, individual properties are further discussed in groups of starches that have similarities in their botanical origin and in groups of starches that have similar properties.

#### Amylose content affects starch digestion kinetics in starches from cereal origin

The observation that amylose content, a high loading on PC2, is not associated with starch digestion kinetics, contradicts with previous research [13–16]. However, these previous studies were performed with starches from cereal origin only, whereas in this study starches from other botanical origins were also included. Therefore, PCA analysis and Pearson’s correlation analysis were repeated with a subdataset that contained data from cereal starches only (Additional file 2: Table S2 and S3). Those analysis revealed a correlation (*r* = − 0.72, *P* = 0.03) between amylose content and starch digestion rate. However, within these cereal starches, interrelationships between amylose content, pores, type of crystalline structure, and the side-chain length distribution of amylopectin were present as well, which were absent in the complete dataset. Those interrelationships between starch characteristics make it impossible to separate effects of the amylose content from other starch characteristics and to draw conclusions on the role of amylose on digestion kinetics.

We conclude that an increased amylose content negatively correlates with starch digestion kinetics when comparing starches within botanic sources, but is not the largest factor in explaining variation in starch digestion kinetics across botanic sources. This is clearly illustrated by a lower (*P* < 0.01) starch hydrolysis of waxy corn (36%) compared with barley (45%) and wheat starch (53%) after 20 min of in vitro digestion. Barley, wheat and waxy corn starch have comparable characteristics, except for the proportion of amylose and the number of pores, as waxy corn starch has a lower amylose content and more pores than barley and wheat starch. This indicates that, across botanic sources, a starch with a low amylose content is not necessarily more rapidly digestible than starches with a normal amylose content.

#### The number of pores does not unambiguously predict variation in starch digestion kinetics

In general, pores are believed to enable enzymes to digest starch granules from the inside out, which increases the rate of hydrolysis of the granules [12, 28]. However, several starch samples with no pores or a low number of pores (barley and wheat) reached a higher (*P* < 0.0001) starch hydrolysis after 20 min of in vitro digestion (45% and 53%, respectively) than corn A (25%), which has many pores (Table 1). Previous studies reporting positive correlations between pores and digestion kinetics, mainly focused on corn [12, 28]. Indeed, within our corn starches, the number of pores is also positively associated with *K* (*r* = 0.94, *P* = 0.0173). However, correlations between the number of pores and the amylose content (*r* = − 0.95, *P* = 0.0324), and number of pores and the percentage of A-type crystals (*r* = 0.89, *P* = 0.0004) are also observed within these samples and have additionally been shown for barley starch previously [46]. Therefore, we conclude that even though the number of pores is associated with other starch properties, it may causally explain variation in starch digestion kinetics within a botanic source, but not across botanic sources.

#### Granule diameter alone does not predict variation in starch digestion kinetics

A correlation between the starch granule diameter and *K* value was identified (*r* = − 0.57, *P* = 0.03), when analysing the complete set of starches. However, it is not clear whether this correlation is the result of collinearity between the granule diameter and another starch parameter, or whether the granular size is affecting the digestion rate. Consequently, additional statistical analysis of sieved fractions of potato starch provides was performed. Those five sieved starch fractions differ primarily in average granule diameters, varying from 18.9 μm to 92.2 μm, whereas the difference among the other properties was negligible (Table 1). Within these sieved fractions, granule diameter was not significantly correlated with *K* (*r* = − 0.65, *P* = 0.2307), demonstrating that for the potato starch used in this study, granule diameter, and consequently surface area, has no causal relation with digestion kinetics. Previous research on the relation between granule diameter and digestion kinetics showed interrelationships between variables such as granule diameter and amylose content [26, 47] and the ability of α-amylase to adhere to granules [48]. This implies that granule diameter and surface area are not causally related to starch digestion kinetics.

#### The type of crystalline structure and chain length distribution of amylopectin explain variation in digestion kinetics across botanic sources

**Fig. 7:**
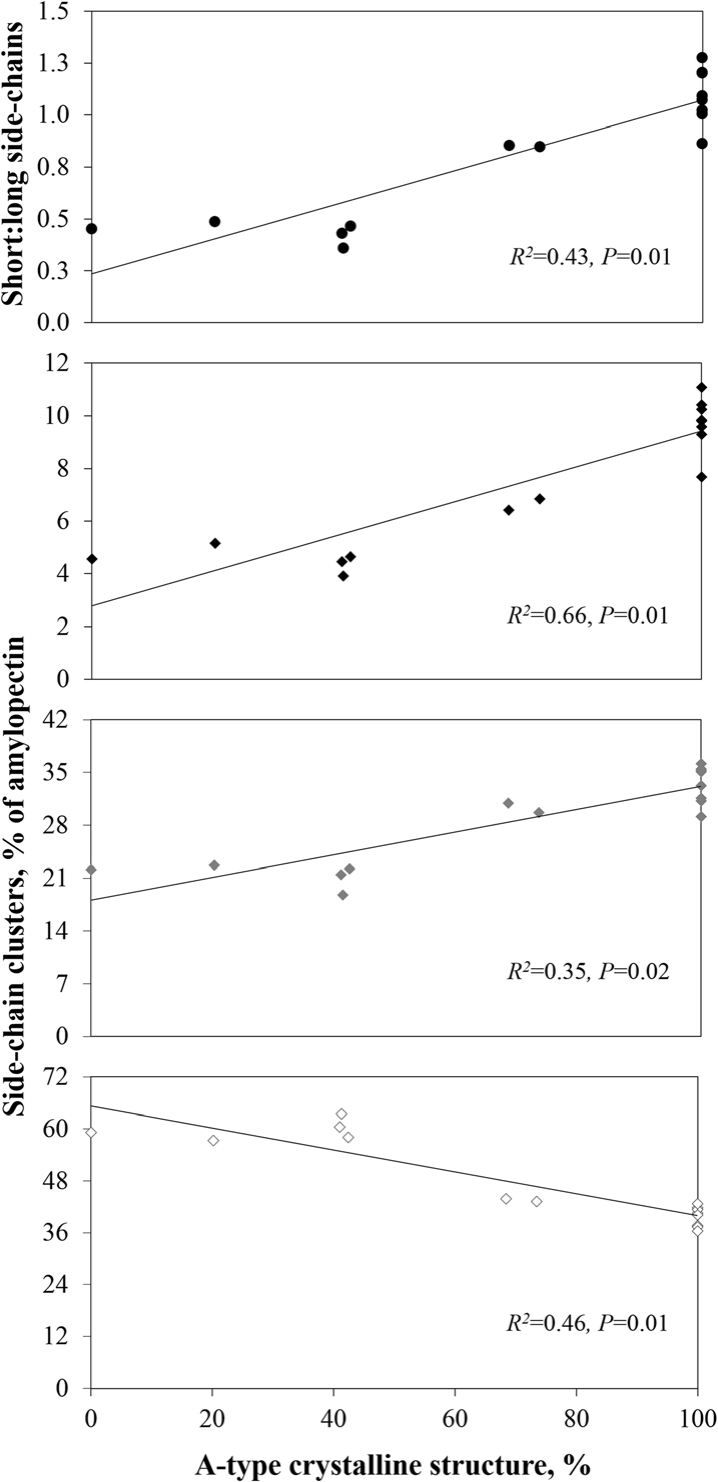
Relation between type of crystalline structure and ratio of short:long amylopectin side-chains (●) and side-chain length clusters DP 6–12 (♦), DP 13–24 (), and DP > 36 () within all (non-sieved) starch samples

After eliminating the granule diameter and number of pores as causal factors affecting starch digestion kinetics across botanic sources, only the percentage of A-type crystals and ratio of short (DP < 24) to long (DP >  36) amylopectin side-chains remain as starch characteristics that affect starch digestion kinetics. Both show strong positive correlations with *K* (*r* = 0.81, *P* = 0.0003; *r* = 0.65, *P* = 0.0081 respectively) but they also have a strong correlation with each other (Fig. 7), confirming previous data [14, 20–23, 40, 42]. In general, low and normal amylose cereal starches consist of both A-type crystals and a high ratio of short:long amylopectin side-chains, whereas potato starch displays B-type crystals and longer amylopectin side chains, and pea and high amylose cereal starches have intermediate crystalline types and amylopectin chain length distributions. We therefore conclude that these are the only measured characteristics that explain variation in digestion kinetics among botanic sources.

#### Characteristics that additionally explain variation in starch digestion depend on the botanic origin

The proportion of variance (*R*^2^) in digestion kinetics explained by PC1 across all botanic sources did not exceed 45% and was lower than the proportion of variance explained by the type of crystalline structure alone (*R*^2^ = 66%). In an attempt to further challenge the concept of additivity, interrelations between the measured starch properties were examined within several clusters of botanic sources and illustrated with PCA. The structural starch properties were correlated with in vitro digestion kinetics within those clusters of botanic sources. When exploring the correlations between starch properties, the lack of additivity proved to be highly affected by the combination of selected botanic sources that were evaluated statistically. For example, multivariate analysis with only corn, pea and potato starches resulted in an PC loading profile that is comparable with the one obtained from the full dataset (Additional file 3: Table S4). However, instead of 45%, 93% of the variation in starch digestion kinetics within this botanic cluster can be explained with this combination of starch characteristics. The combination of characteristics also additively explains variation, as *R*^2^ for PC1 (93%) exceeds that of the type of crystalline structure (81%), the number of pores (89%), and the ratio of short: long amylopectin side chains (87%) individually (Additional file 3: Table S5). Alternatively, there may be a role for other starch properties in explaining starch digestion behaviour, which were not included in this study. For example, previous studies proved that digestion kinetics can also be affected by minor components present such as proteins, lipids and phosphorus, which make up < 1.5% of the starch granule [6] and by variation in amylose structure [49]. However, based on literature, we believe that the selection of starch characteristics made for this study, covers the most important variation in molecular and structural properties of the starch granule. The lack of additivity of those starch properties in relation to variation in starch digestion kinetics, indicates that variation in most starch properties explains variation within, rather than among botanic sources.

## Conclusions

Across all analysed botanic sources, the type of crystalline structure and the amylopectin side-chain length distribution explains most variation in in vitro digestibility kinetics among starches commonly used in pig nutrition. Granule size is not causally related to starch digestion kinetics, and amylose content and number of pores appeared to explain variation within rather than across botanical sources. Furthermore, within (clusters of) botanical sources variation in digestion kinetics is additively explained by other starch properties measured.

## Additional files

Additional file 1: **Table S1.** In vitro digestion of all analysed starches. (DOCX 36 kb)

Additional file 2: **Table S2.** Rotated factor pattern, eigenvalues and proportion variance explained of principle components in multivariate analysis of subdataset 1^1^. **Table S3.** Pearson correlation coefficients for starch properties and digestion kinetics, within subdataset 1^1,2^. (DOCX 36 kb)

Additional file 3: **Table S4.** Rotated factor pattern, eigenvalues and proportion variance explained by principle components in multivariate analysis of subdataset 2^1^. **Table S5.** Pearson correlation coefficients for starch properties and digestion kinetics, within subdataset 2^1,2^. (DOCX 36 kb)

## Abbreviations

DP: Degree of polymerization
*K*: In vitro rate constant of starch hydrolysed to glucose per minute
PC: Principal Component
PCA: Principal Component Analysis
RDS: Rapidly digestible starch
RS: Resistant starch
SDS: Slowly digestible starch

## References

[1] Da Silva CS, Bosch G, Bolhuis J, Stappers L, van Hees H, Gerrits W (2014). Effects of alginate and resistant starch on feeding patterns, behaviour and performance in ad libitum-fed growing pigs. Animal.

[2] Bolhuis J, Van den Brand H, Staals S, Zandstra T, Alferink S, Heetkamp M (2008). Effects of fermentable starch and straw-enriched housing on energy partitioning of growing pigs. Animal.

[3] van den Borne JJGC, Schrama JW, Heetkamp MJW, Verstegen MWA, Gerrits WJJ (2007). Synchronising the availability of amino acids and glucose increases protein retention in pigs. Animal.

[4] Weurding R, Enting H, Verstegen M (2003). The relation between starch digestion rate and amino acid level for broiler chickens. Poult Sci.

[5] Tester RF, Karkalas J, Qi X (2004). Starch—composition, fine structure and architecture. J Cereal Sci.

[6] Robyt JF, Fraser-Reid BO, Tatsuta K, Thiem J (2008). Starch: structure, properties, chemistry, and enzymology. Glycoscience.

[7] Gallant DJ, Bouchet B, Baldwin PM (1997). Microscopy of starch: evidence of a new level of granule organization. Carbohydr Polym.

[8] Hizukuri S (1986). Polymodal distribution of the chain lengths of amylopectins, and its significance. Carbohydr Res.

[9] Robin J, Mercier C, Charbonniere R, Guilbot A (1974). Lintnerized starches. Gel filtration and enzymatic studies of insoluble residues from prolonged acid treatment of potato starch. Cereal Chem.

[10] Hanashiro I, J-i A, Hizukuri S (1996). A periodic distribution of the chain length of amylopectin as revealed by high-performance anion-exchange chromatography. Carbohydr Res.

[11] Jane J, Chen Y, Lee L, McPherson A, Wong K, Radosavljevic M (1999). Effects of amylopectin branch chain length and amylose content on the gelatinization and pasting properties of starch. Cereal Chem.

[12] Fannon JE, Hauber RJ, BeMiller JN (1992). Surface pores of starch granules. Cereal Chem.

[13] Asare EK, Jaiswal S, Maley J, Baga M, Sammynaiken R, Rossnagel BG (2011). Barley grain constituents, starch composition, and structure affect starch in vitro enzymatic hydrolysis. J Agric Food Chem.

[14] Kong X, Chen Y, Zhu P, Sui Z, Corke H, Bao J (2015). Relationships among genetic, structural, and functional properties of Rice starch. J Agric Food Chem.

[15] Shu X, Jia L, Ye H, Li C, Wu D (2009). Slow digestion properties of rice different in resistant starch. J Agric Food Chem.

[16] Li Y, Zhang AR, Luo HF, Wei H, Zhou Z, Peng J (2015). In vitro and in vivo digestibility of corn starch for weaned pigs: effects of amylose:amylopectin ratio, extrusion, storage duration, and enzyme supplementation. J Anim Sci.

[17] Goddard MS, Young G, Marcus R (1984). The effect of amylose content on insulin and glucose responses to ingested rice. Am J Clin Nutr.

[18] Granfeldt Y, Liljeberg H, Drews A, Newman R, Bjorck I (1994). Glucose and insulin responses to barley products: influence of food structure and amylose-amylopectin ratio. Am J Clin Nutr.

[19] Trinidad TP, Mallillin AC, Encabo RR, Sagum RS, Felix AD, Juliano BO (2013). The effect of apparent amylose content and dietary fibre on the glycemic response of different varieties of cooked milled and brown rice. Int J Food Sci Nutr.

[20] Hizukuri S, Kaneko T, Takeda Y (1983). Measurement of the chain length of amylopectin and its relevance to the origin of crystalline polymorphism of starch granules. Biochim Biophys Acta.

[21] Zhang G, Ao Z, Hamaker BR (2008). Nutritional property of endosperm starches from maize mutants: a parabolic pelationship between slowly digestible starch and amylopectin fine structure. J Agric Food Chem.

[22] Regmi PR, van Kempen TA, Matte JJ, Zijlstra RT (2011). Starch with high amylose and low in vitro digestibility increases short-chain fatty acid absorption, reduces peak insulin secretion, and modulates incretin secretion in pigs. J Nutr.

[23] Jiang H, Jane JL, Acevedo D, Green A, Shinn G, Schrenker D (2010). Variations in starch physicochemical properties from a generation-means analysis study using amylomaize V and VII parents. J Agric Food Chem.

[24] Shrestha AK, Blazek J, Flanagan BM, Dhital S, Larroque O, Morell MK (2012). Molecular, mesoscopic and microscopic structure evolution during amylase digestion of maize starch granules. Carbohydr Polym.

[25] Faisant N, Champ M, Colonna P, Buleon A, Molis C, Langkilde AM (1993). Structural features of resistant starch at the end of the human small intestine. Eur J Clin Nutr.

[26] Franco CM, do Rio Preto SJ, Ciacco CF (1992). Factors that affect the enzymatic degradation of natural starch granules-effect of the size of the granules. Starch-Stärke..

[27] Planchot V, Colonna P, Gallant DJ, Bouchet B (1995). Extensive degradation of native starch granules by alpha-amylase from aspergillus fumigatus. J Cereal Sci.

[28] Kong BW, Kim JI, Kim MJ, Kim JC (2003). Porcine pancreatic alpha-amylase hydrolysis of native starch granules as a function of granule surface area. Biotechnol Prog.

[29] Hoebler C, Karinthi A, Chiron H, Champ M, Barry J (1999). Bioavailability of starch in bread rich in amylose: metabolic responses in healthy subjects and starch structure. Eur J Clin Nutr.

[30] Cheetham NWH, Tao L (1998). Variation in crystalline type with amylose content in maize starch granules: an X-ray powder diffraction study. Carbohydr Polym.

[31] Wei C, Xu B, Qin F, Yu H, Chen C, Meng X (2010). C-type starch from high-amylose rice resistant starch granules modified by antisense RNA inhibition of starch branching enzyme. J Agric Food Chem.

[32] Stevnebø A, Sahlström S, Svihus B (2006). Starch structure and degree of starch hydrolysis of small and large starch granules from barley varieties with varying amylose content. Anim Feed Sci Technol.

[33] Tester R, Qi X, Karkalas J (2006). Hydrolysis of native starches with amylases. Anim Feed Sci Technol.

[34] Englyst HN, Kingman SM, Cummings JH (1992). Classification and measurement of nutritionally important starch fractions. Eur J Clin Nutr.

[35] van Kempen TA, Regmi PR, Matte JJ, Zijlstra RT (2010). In vitro starch digestion kinetics, corrected for estimated gastric emptying, predict portal glucose appearance in pigs. J Nutr.

[36] Zobel H (1988). Starch crystal transformations and their industrial importance. Starch - Stärke.

[37] Jane JL, Kasemsuwan T, Leas S, Zobel H, Robyt JF (1994). Anthology of starch granule morphology by scanning electron microscopy. Starch-Stärke.

[38] Buléon A, Colonna P, Planchot V, Ball S (1998). Starch granules: structure and biosynthesis. Int J Biol Macromol.

[39] Shi YC, Seib PA (1992). The structure of four waxy starches related to gelatinization and retrogradation. Carbohydr Res.

[40] Witt T, Doutch J, Gilbert EP, Gilbert RG (2012). Relations between molecular, crystalline, and lamellar structures of amylopectin. Biomacromolecules.

[41] Hoover R, Vasanthan T (1994). Effect of heat-moisture treatment on the structure and physicochemical properties of cereal, legume, and tuber starches. Carbohydr Res.

[42] Wang K, Henry R, Gilbert R (2014). Causal relations among starch biosynthesis, structure, and properties. Springer Sci Rev.

[43] Kalichevsky MT, Orford PD, Ring SG (1990). The retrogradation and gelation of amylopectins from various botanical sources. Carbohydr Res.

[44] Juszczak L, Fortuna T, Wodnicka K (2002). Characteristics of cereal starch granules surface using nitrogen adsorption. J Food Eng.

[45] van Kempen TA, Pujol S, Tibble S, Balfagon A. In vitro characterization of starch digestion and its implications for pigs. In: Wiseman J, Varley MA, McOrist S, Kemp B, editors. 62nd Easter School in the Agricultural and Food Sciences; Paradigms in pig science. Nottingham: University of Nottingham; 2007. p. 515–25.

[46] Li JH, Vasanthan T, Hoover R, Rossnagel BG (2004). Starch from hull-less barley: V. in-vitro susceptibility of waxy, normal, and high-amylose starches towards hydrolysis by alpha-amylases and amyloglucosidase. Food Chem.

[47] Knutson C, Khoo U, Cluskey J, Inglett G (1982). Variation in enzyme digestibility and gelatinization behavior of corn starch granule fractions. Cereal Chem.

[48] MacGregor A (1979). Isolation of large and small granules of barley starch and a study of factors influencing the adsorption of barley malt alpha-amylase by these granules. Cereal Chem.

[49] Yu W, Tao K, Gilbert RG (2018). Improved methodology for analyzing relations between starch digestion kinetics and molecular structure. Food Chem.

