# Supplementary material for: Amylopectin structure and crystallinity explains variation in digestion kinetics of starches across botanic sources in an in vitro pig model
Source: J Anim Sci Biotechnol. 2018 Dec 29;9:91. doi: 10.1186/s40104-018-0303-8 (PMC6310989; doi:10.1186/s40104-018-0303-8)
Supplement: Supplementary file 1 — Table S1. In vitro digestion of all analysed starches. (DOCX 36 kb) [file 40104_2018_303_MOESM1_ESM.docx]

Table S 1. In vitro digestion of all analysed starches

| **Origin** | **sample** | **Average cumulative starch hydrolysis ± standard deviation, %** | | | | | | | |
| --- | --- | --- | --- | --- | --- | --- | --- | --- | --- |
|  |  | 0 min | 10 min | 20 min | 60 min | 120 min | 180 min | 240 min | 360 min |
| Potato | Regular | -1±0.6 | 1±0.1 | -1±2.2 | 4±2.0 | 12±1.9 | 17±2.4 | 22±3.1 | 32±6.1 |
| Potato | Sieved (<20) | 0±0.0 | 2±0.5 | 4±0.5 | 8±0.3 | 17±1.7 | 21±0.6 | 28±0.9 | 40±2.7 |
| Potato | Sieved (21-32) | 0±0.0 | 0±0.4 | 2±0.5 | 5±0.3 | 11±0.1 | 16±1.0 | 21±1.0 | 31±2.8 |
| Potato | Sieved (33-52) | 0±0.1 | 1±0.8 | 2±0.8 | 4±0.8 | 8±1.0 | 12±1.5 | 17±1.3 | 25±2.5 |
| Potato | Sieved (53-71) | 0±0.1 | 1±0.6 | 2±0.6 | 4±0.9 | 9±0.9 | 13±0.6 | 18±1.3 | 26±2.8 |
| Potato | Sieved (72-109) | -1±1.0 | 0±0.8 | 2±0.5 | 5±0.2 | 10±0.8 | 15±0.9 | 20±0.9 | 28±2.3 |
| Potato | Waxy | 0±0.1 | 2±0.1 | 3±0.2 | 8±0.2 | 13±0.9 | 18±0.5 | 22±1.2 | 28±0.6 |
| Potato | HMT | 0±0.1 | 3±1.2 | 6±0.3 | 21±1.0 | 39±1.2 | 53±1.6 | 63±1.5 | 78±0.7 |
| Pea | Regular A | 0±0.7 | 4±3.9 | 10±3.9 | 33±4.9 | 55±5.2 | 69±5.5 | 75±6.6 | 86±7.3 |
| Pea | Regular B | 0±0.1 | 5±2.2 | 10±1.9 | 32±3.5 | 56±1.1 | 73±1.5 | 81±2.7 | 90±1.3 |
| Corn | Waxy | -1±0.6 | 21±1.7 | 36±3 | 81±2.0 | 98±1.3 | 102±5.3 | 100±5.3 | 99±7.2 |
| Corn | Regular A | 0±0.1 | 11±3.2 | 25±1.3 | 70±3.6 | 95±4.5 | 101±1.2 | 103±1.6 | 106±3.2 |
| Corn | Regular B | 0±0.3 | 14±1.1 | 27±1.1 | 72±1.9 | 98±1.7 | 103±2.1 | 102±4.0 | 103±2.3 |
| Corn | High amylose A | 0±0.6 | 6±1.3 | 6±4.6 | 21±3.1 | 34±3.5 | 44±1.5 | 50±3.7 | 57±4.9 |
| Corn | High amylose B | 0±0.4 | 5±0.5 | 9±0.5 | 20±1.3 | 34±0.9 | 45±2.1 | 51±2.5 | 60±2.3 |
| Barley | Regular | 0±0.2 | 22±2.6 | 45±3.1 | 93±5.6 | 100±3.1 | 100±4.9 | 101±4.2 | 100±2.6 |
| Wheat | Regular | 0±0.3 | 34±2.4 | 57±2.5 | 94±0.5 | 99±1.0 | 101±3.2 | 101±3.4 | 100±1.1 |
| Rice | Waxy | 0±0.3 | 36±2.9 | 53±3.6 | 95±4.2 | 101±2.3 | 102±2.9 | 101±3.3 | 102±3.5 |
| Rice | Regular A | 0±0.2 | 32±2.5 | 42±1.5 | 81±2.5 | 95±2.6 | 94±3.0 | 95±2.5 | 96±0.7 |
| Rice | Regular B | 0±0.5 | 18±3.8 | 27±2.7 | 61±3.4 | 81±2.5 | 90±4.7 | 94±3.0 | 96±3.2 |
