# Supplementary material for: Amylopectin structure and crystallinity explains variation in digestion kinetics of starches across botanic sources in an in vitro pig model
Source: J Anim Sci Biotechnol. 2018 Dec 29;9:91. doi: 10.1186/s40104-018-0303-8 (PMC6310989; doi:10.1186/s40104-018-0303-8)
Supplement: Supplementary file 2 — Table S2. Rotated factor pattern, eigenvalues and proportion variance explained of principle components in multivariate analysis of subdataset 11. Table S3. Pearson correlation coefficients for starch properties and digestion kinetics, within subdataset 11,2. (DOCX 36 kb) [file 40104_2018_303_MOESM2_ESM.docx]

Table S 2. Rotated factor pattern, eigenvalues and proportion variance explained of principle components in multivariate analysis of subdataset 1^1^.

|  | **PC1** | **PC2** |
| --- | --- | --- |
| Eigenvalues | 3.58 | 1.11 |
| Proportion variance explained | 0.60 | 0.19 |
| Loading of variables | | |
| Granule diameter | 0.03 | 0.97 |
| Number of pores | 0.72 | -0.28 |
| Crystal content | -0.71 | -0.22 |
| Percentage A-type crystals | 0.91 | -0.07 |
| Amylose content | -0.91 | 0.16 |
| Ratio short:long amylopectin side-chains | 0.95 | 0.16 |

^1^ Subdataset 1 contains only data from cereal starches.

Table S 3. Pearson correlation coefficients for starch properties and digestion kinetics, within subdataset 1^1,2^.

|  | | **PC1** | **PC2** | **Amylose content** | **Granule diameter** | **Crystal content** | **% A-type crystals** | **Number of pores** | **Side-chain length amylopectin, DP** | | | | | **K** |
| --- | --- | --- | --- | --- | --- | --- | --- | --- | --- | --- | --- | --- | --- | --- |
|  |  |  |  |  |  |  |  |  | **6 – 12** | **13 – 24** | **25 – 36** | **> 36** | **Short:long^3^** |  |
| **PC1** | | X | 0.00 | -0.91** | -0.03 | -0.71** | 0.91** | 0.72** | 0.93** | 0.97** | 0.43 | -0.95** | 0.95** | 0.56* |
| **PC2** | |  | X | 0.16 | 0.97** | -0.22 | -0.07 | -0.28 | -0.10 | 0.08 | 0.58** | -0.22 | 0.046 | -0.21 |
| **Amylose content** | |  |  | X | 0.09 | 0.46 | -0.88** | -0.59* | -0.87** | -0.83** | -0.44 | 0.85** | -0.82** | -0.72** |
| **Granule diameter** | |  |  |  | X | -0.12 | -0.06 | -0.13 | -0.09 | 0.10 | 0.60* | -0.23 | 0.19 | -0.17 |
| **Crystalline content** | |  |  |  |  | X | -0.58* | -0.41 | -0.63* | -0.69** | -0.19 | 0.64** | -0.64** | -0.27 |
| **% A-type crystals** | |  |  |  |  |  | X | 0.43 | 0.90** | 0.87** | 0.65** | -0.94** | 0.86** | 0.75** |
| **Number of pores** | |  |  |  |  |  |  | X | 0.51 | 0.68** | 0.00 | -0.56* | 0.66** | -0.06 |
| **Side-chain length amylopectin, DP** | **6 - 12** |  |  |  |  |  |  |  | X | 0.94** | 0.42 | -0.95** | 0.95** | 0.71** |
|  | **13 - 24** |  |  |  |  |  |  |  |  | X | 0.40 | -0.97** | 0.99** | 0.49 |
|  | **25 - 36** |  |  |  |  |  |  |  |  |  | X | -0.60* | 0.45 | 0.63* |
|  | **> 36** |  |  |  |  |  |  |  |  |  |  | X | -0.98** | -0.64** |
|  | **Short:long** |  |  |  |  |  |  |  |  |  |  |  | X | 0.52* |
| **K** | |  |  |  |  |  |  |  |  |  |  |  |  | X |

^1^ Subdataset 1 contains only data from cereal starches.

^2^ ** indicates a significant correlation (*P*≤0.05), * indicates a tendency for a significant correlation (0.05< *P*≤0.10)

^3^”Short” refers to amylopectin side-chains with DP 6-24 and “long” refers to amylopectin side-chains with DP
