# Supplementary material for: Amylopectin structure and crystallinity explains variation in digestion kinetics of starches across botanic sources in an in vitro pig model
Source: J Anim Sci Biotechnol. 2018 Dec 29;9:91. doi: 10.1186/s40104-018-0303-8 (PMC6310989; doi:10.1186/s40104-018-0303-8)
Supplement: Supplementary file 3 — Table S4. Rotated factor pattern, eigenvalues and proportion variance explained by principle components in multivariate analysis of subdataset 21. Table S5. Pearson correlation coefficients for starch properties and digestion kinetics, within subdataset 21,2. (DOCX 36 kb) [file 40104_2018_303_MOESM3_ESM.docx]

Table S 4. Rotated factor pattern, eigenvalues and proportion variance explained by principle components in multivariate analysis of subdataset 2^1^.

|  | **PC1** | **PC2** |
| --- | --- | --- |
| Eigenvalues | 3.18 | 1.45 |
| Proportion variance explained | 0.53 | 0.24 |
| Loading on PC of each variable | | |
| Granule diameter | -0.62 | -0.69 |
| Number of pores | 0.88 | -0.14 |
| Crystal content | -0.45 | 0.51 |
| Percentage A-type crystals | 0.96 | 0.09 |
| Amylose content | -0.10 | 0.80 |
| Ratio short:long amylopectin side-chains | 0.94 | -0.21 |

^1^ Subdataset 2 contains only data from corn, pea and potato starches.

Table S 5. Pearson correlation coefficients for starch properties and digestion kinetics, within subdataset 2^1,2^.

|  | | **PC1** | **PC2** | **Amylose content** | **Granule diameter** | **Crystal content** | **% A-type crystals** | **Number of pores** | **Side-chain length amylopectin, DP** | | | | | **K** |
| --- | --- | --- | --- | --- | --- | --- | --- | --- | --- | --- | --- | --- | --- | --- |
|  |  |  |  |  |  |  |  |  | **6 – 12** | **13 – 24** | **25 – 36** | **> 36** | **Short:long^3^** |  |
| **PC1** | | X | 0.00 | -0.10 | -0.62** | -0.45* | 0.96** | 0.88** | 0.93** | 0.90** | 0.72* | -0.91* | 0.94** | 0.97** |
| **PC2** | |  | X | 0.80** | -0.69** | 0.51* | 0.09 | -0.14 | -0.24 | -0.29 | -0.07 | 0.24 | -0.21 | -0.08 |
| **Amylose content** | |  |  | X | -0.32 | 0.11 | 0.02 | -0.26 | -0.30 | -0.31 | -0.14 | 0.28 | -0.27 | -0.21 |
| **Granule diameter** | |  |  |  | X | -0.14 | -0.63** | -0.44* | -0.38 | -0.34 | -0.42 | 0.38 | -0.41 | -0.53** |
| **Crystalline content** | |  |  |  |  | X | -0.43 | -0.30 | -0.45* | -0.52** | -0.39 | 0.50* | -0.46* | -0.35 |
| **% A-type crystals** | |  |  |  |  |  | X | 0.76** | 0.87** | 0.85** | 0.78** | -0.88** | 0.88** | 0.90** |
| **Number of pores** | |  |  |  |  |  |  | X | 0.85** | 0.77** | 0.41 | -0.74** | 0.82** | 0.94** |
| **Side-chain length amylopectin, DP** | **6 - 12** |  |  |  |  |  |  |  | X | 0.97** | 0.70** | -0.96** | 0.99** | 0.94** |
|  | **13 - 24** |  |  |  |  |  |  |  |  | X | 0.80** | -0.99** | 0.99** | 0.89** |
|  | **25 - 36** |  |  |  |  |  |  |  |  |  | X | -0.86** | 0.77** | 0.62** |
|  | **> 36** |  |  |  |  |  |  |  |  |  |  | X | -0.99** | -0.88** |
|  | **Short:long** |  |  |  |  |  |  |  |  |  |  |  | X | 0.93** |
| **K** | |  |  |  |  |  |  |  |  |  |  |  |  | X |

^1^ Subdataset 2 contains only data from corn, pea and potato starches.

^2^ ** indicates a significant correlation (*P*≤0.05), * indicates a tendency for a significant correlation (0.05< *P*≤0.10).

^3^”Short” refers to amylopectin side-chains with DP 6-24 and “long” refers to amylopectin side-chains with DP>36.
